# Prognostic value of low CDX2 expression in colorectal cancers with a high stromal content – a short report

**DOI:** 10.1007/s13402-019-00436-0

**Published:** 2019-03-08

**Authors:** Tessa P. Sandberg, Iris Sweere, Gabi W. van Pelt, Hein Putter, Louis Vermeulen, Peter J. Kuppen, Rob A. E. M. Tollenaar, Wilma E. Mesker

**Affiliations:** 1 Department of Surgery, Leiden University Medical Centrum, Albinusdreef 2, 2333ZA Leiden, The Netherlands; 2 Department of Pathology, Leiden University Medical Centrum, Leiden, The Netherlands; 3 Department of Medical Statistics, Leiden University Medical Centrum, Leiden, The Netherlands; 4 Laboratory for Experimental Oncology and Radiobiology (LEXOR), Center for Experimental and Molecular Medicine (CEMM), Academic Medical Center & Cancer Center Amsterdam, Amsterdam, The Netherlands

**Keywords:** Colorectal cancer, CDX2 expression, Tumour-stroma ratio, Prognosis

## Abstract

**Purpose:**

Lack of expression of the intestinal transcription factor CDX2 in colorectal cancer (CRC) identifies patients with a poor prognosis. This biomarker has previously been suggested to be prognostic in CRCs with a high stromal content based on mRNA expression data. We investigated the prognostic value of CDX2 expression in microsatellite stable CRC stratified by stromal content using microscopy-based techniques.

**Methods and results:**

The study included a cohort of 236 patients with stage I-IV CRC. We assessed by microscopy the tumour-stroma ratio (TSR) and the immunohistochemical CDX2 intensity. We found that patients of the stroma-high group had a worse prognosis compared to those of the stroma-low group [disease-free survival in a multivariate analysis (DFS_multivariate_) HR 1.52 (95% CI 1.05–2.21)]. In our cohort, low CDX2 expression (14.6%) showed prognostic value for DFS_multivariate_ [HR 1.93 (95% CI 1.16–3.23)]. Interestingly, when stratifying the cohort by TSR, no prognostic difference was observed related to CDX2 expression in stroma-low tumours. However, CDX2 expression was found to be prognostic within the stroma-high group [DFS_multivariate_ HR 3.02 (95% CI 1.49–6.13)]. The *p* value for interaction between TSR and CDX2 status was borderline significant in DFS (*p* = 0.071).

**Conclusions:**

The present study confirms a poor outcome of patients with stroma-high tumours. Low CDX2 expression in tumours with a high stromal content identified patients with a particularly poor prognosis. The present study did not reveal a clear difference in TSR associated with CDX2 status and survival. This method, solely based on microscopy, identifies patients who have a high risk of relapse and a poor outcome, and who may benefit from targeted therapy.

**Electronic supplementary material:**

The online version of this article (10.1007/s13402-019-00436-0) contains supplementary material, which is available to authorized users.

## Introduction

The transcription factor caudal type homeobox 2 (CDX2) is expressed in intestinal epithelial cells and is considered to act as a tumour suppressor. Several studies have demonstrated that lack of CDX2 expression in colorectal cancer (CRC) is associated with an aggressive behaviour [1–5]. Through a transcriptomic analysis, Pilati et al. [6] found that lack of CDX2 expression was prognostic in consensus molecular subtype 4 (CMS4) tumours, a subtype characterised by a high stromal content, and not in CMS1, a subtype known to be enriched for microsatellite instable high (MSI-H) tumours. Standard pathological assessment relies heavily on microscopic analysis given the current high cost of transcriptomic data acquisition. Therefore, we investigated the prognostic value of the CDX2 status in microsatellite stable (MSS) CRCs stratified by stromal content using solely microscopic techniques. The tumour-stroma ratio (TSR) is commonly used to identify tumours with a high stromal content in conventionally haematoxylin and eosin (H&E)-stained paraffin sections at the invasive part of the tumour [7–9]. We hypothesised that a low CDX2 expression may be prognostic in MSS colorectal tumours with a high stromal content.

## Material and methods

### Patient cohort

The Leiden University Medical centre (LUMC) retrospective study population [10, 11] comprises 236 patients with stage I – IV CRC treated at the LUMC between 1991 and 2001 without neoadjuvant therapy according to the Dutch guidelines of that time (Supplementary Table 1). The inclusion and exclusion criteria have previously been described [10] and patients omitted in the final cohort are depicted in Supplementary Fig. 1. All samples were coded and handled according to the national ethical guidelines, the Netherlands Code for Proper Secondary Use of Human Tissue, and the study was performed in accordance with the Declaration of Helsinki.

### Tumour-stroma ratio assessment

**Fig. 1:**
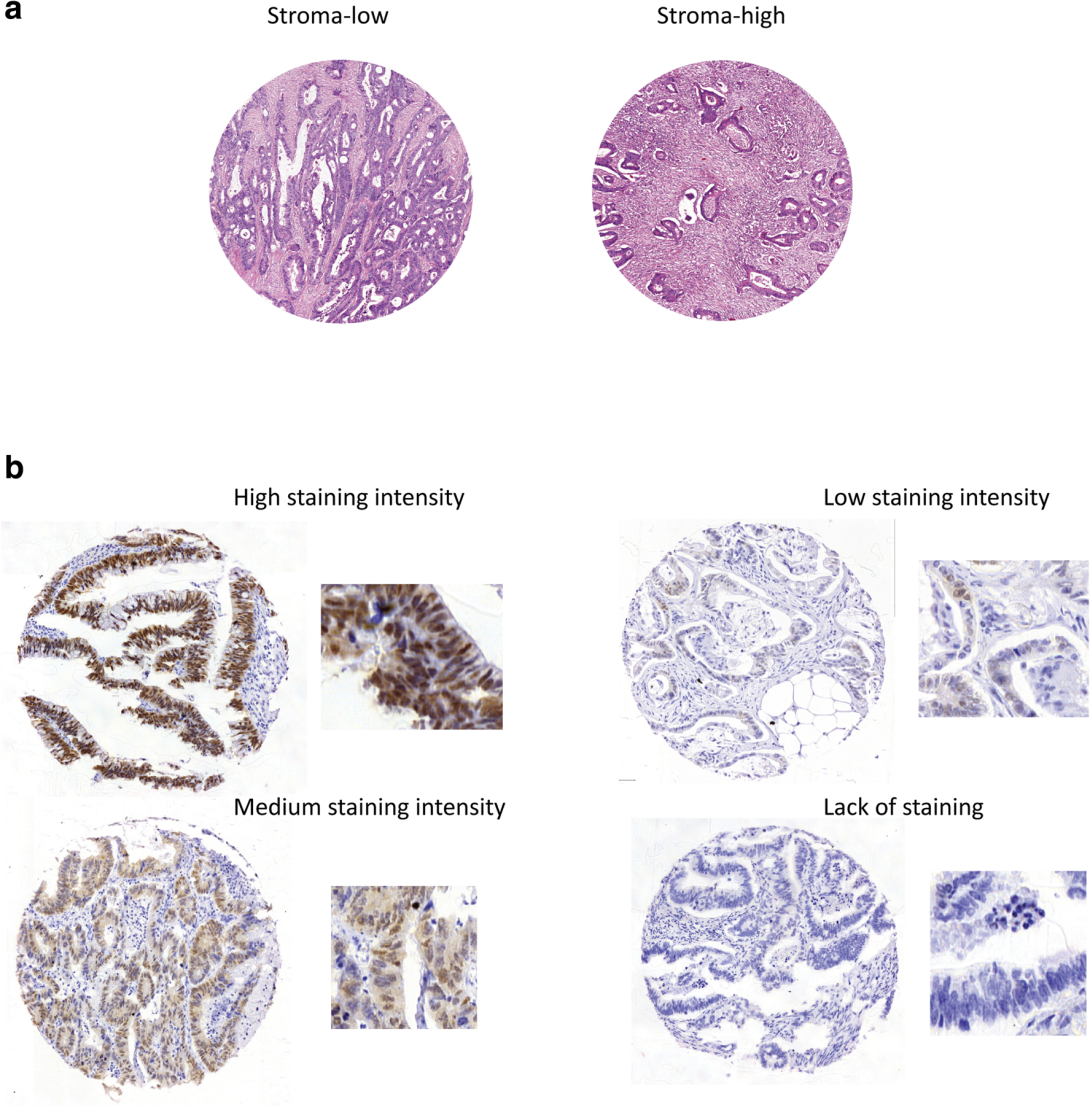
Examples of H&E-stained tissue sections scored for tumour-stroma ratio and cores of tissue microarrays (TMAs) scored for CDX2 expression. Whole H&E-stained tissue sections were scored. The zoomed in regions represent a stroma-low tumour with 20% stroma and the right panel represents a stroma-high tumour with 90% stroma (**a**). Cores of a TMA were scored for CDX2 staining intensity in three categories: CDX2 high, medium and low (**b**). Low staining intensity and lack of CDX2 staining were scored as CDX2 low

Patient material was fixed in formalin and embedded in paraffin. Two investigators independently scored the TSR on the same whole H&E-stained tissue sections from the most invasive part of the primary tumours as described previously [8]. Briefly, the investigators selected and estimated the region with the highest stroma percentage in a 2.5× or 10× microscopic field. A 10× objective microscopic field was scored when tumour cells were present at two opposite borders of the image field. Scoring percentages were given in 10 fold percentage per image field and the final score was assessed in the field with the highest stroma percentage (Fig. 1a). Using a cut-off of 50% as determined previously [12, 13], tumours with ≤ 50% stroma were considered stroma-low and tumours with > 50% stroma were considered stroma-high. The TSR scores were assessed by two investigators (T.P.S and G.W. v.P) in a blinded manner. Both investigators scored 72% of the cohort of which 44% was scored by both investigators. In case of an inconclusive score, a third observer was decisive (W.E. M.).

### CDX2 expression analysis on tissue microarrays

Previously identified [11] MSI-high patients (*N* = 35) were excluded from the final cohort due to their small numbers. The MSS cohort was stained for CDX2 using IHC on tissue microarrays (TMAs) containing 6 μm tumour sections (*N* = 201) [10]. After deparaffinisation and rehydration of the paraffin sections, endogenous peroxidase was blocked and antigen retrieval was performed. Nonspecific protein binding was blocked with 5% goat serum (Sigma). Sections were incubated overnight with an anti-CDX2 antibody (Novus Biologicals, NB100–2136, 1:200). On the following day, the sections were incubated for 30 min with a HRP-labelled anti-rabbit secondary antibody (EnVision^+^, Dako) and visualised using DAB (Dako). A total of 538 tissue cores was used in the study and mostly three cores per patient were evaluated (mean = 2.7, SD = 0.5). As depicted in Fig. 1b, low intensity represents absence or very weak nuclear staining, medium intensity represents a clear drop in nuclear expression and excludes strongly stained nuclei, and high intensity represents a strong nuclear expression comparable to that in healthy colon tissue. The TMA cores also showed diffuse intensity differences between cells. The highest score of the TMA cores of each patient was reported as the final score of the patient. Two researchers (I.S. and T.P.S.) scored the CDX2 staining intensities in a blinded manner. I.S. scored 80% of the cohort and T.P.S. the entire cohort. In case of disagreement, discordant samples were discussed until consensus was reached.

### Statistical analyses

Statistical analyses and figures were made in R (version 3.4.3) using Rstudio (version 1.1.5). Figure compositions were performed in Illustrator Adobe (version 22.0.1). Cohen’s kappa-value was calculated to assess the inter-observer agreement. Disease-free survival (DFS) was defined as the time period between surgery and recurrence, new occurrence of CRC or death from any cause. Overall survival (OS) was defined as the time period between surgery and death from any cause. Patients who were event-free but lost from follow-up were censored at the last date at which they were known to be event-free. Log-rank tests were performed to compare the 5-year survival probabilities between groups. When more than two groups were compared, pairwise comparisons were performed between two groups and the false-discovery rate was adjusted using the Benjamini-Hochberg procedure. Univariate Cox regression analyses for survival were performed to test differences in OS and DFS of each variable. The variables to include in the final model were selected using backward stepwise elimination based on the smallest Akaike information criterion (AIC). The potential variables to include in the model were age (categories), sex, TNM classification (AJCC5), location of the tumour (colon versus rectum) and adjuvant chemotherapy. Differentiation grade was not included due to missing data. Since a low number of events was recorded in stage I, we combined stage I and II to one category. Once the variables were selected, we tested whether the variables met the proportional hazard assumptions using Schoenfeld residuals correlation with time. If the proportional hazard assumption was violated, the variable was stratified over time and the assumption was tested again for violation. Next, the Cox Proportional Hazard regression model was applied. Missing values were not included. An interaction test was performed in the univariate and multivariate analyses. The likelihood-ratio test was used to compare the goodness of fit of the alternative model comprising a cross product of TSR and CDX2 status with the reference model comprising both variables. Lastly, a sensitivity analysis using log-rank test was performed to determine the robustness of the results. Patients with stage IV disease were excluded in this analysis because this subgroup is known to have a particularly poor prognosis. The level of significance was set at *p* < 0.05 unless stated otherwise.

## Results and discussion

The cohort consisted of 201 MSS CRC patients for which the TSR was scored (Supplementary Table 1). The inter-observer agreement was 0.8 (Cohen’s kappa-value). A similar proportion of patients had tumours scored as stroma-low (*N* = 105, 52.2%) and as stroma-high (*N* = 96, 47.8%). After scoring the TMA for CDX2 staining intensity, two patients were excluded from the analysis due to low quality of the TMA cores (*N* = 199). The inter-observer agreement for CDX2 when dividing the cohort into low, medium and high staining intensity was 0.6 (Cohen’s kappa-value). The inter-observer agreement increased to 0.8 when comparing low versus medium and high CDX2 staining intensities (Cohen’s kappa-value). In total 118 (59.3%) patients were classified into a CDX2 high group and 52 (26.1%) into a CDX2 medium group. A minority of 29 patients (14.6%) exhibited a lack of or a low CDX2 expression (Fig. 1, Supplementary Table 1).

**Fig. 2:**
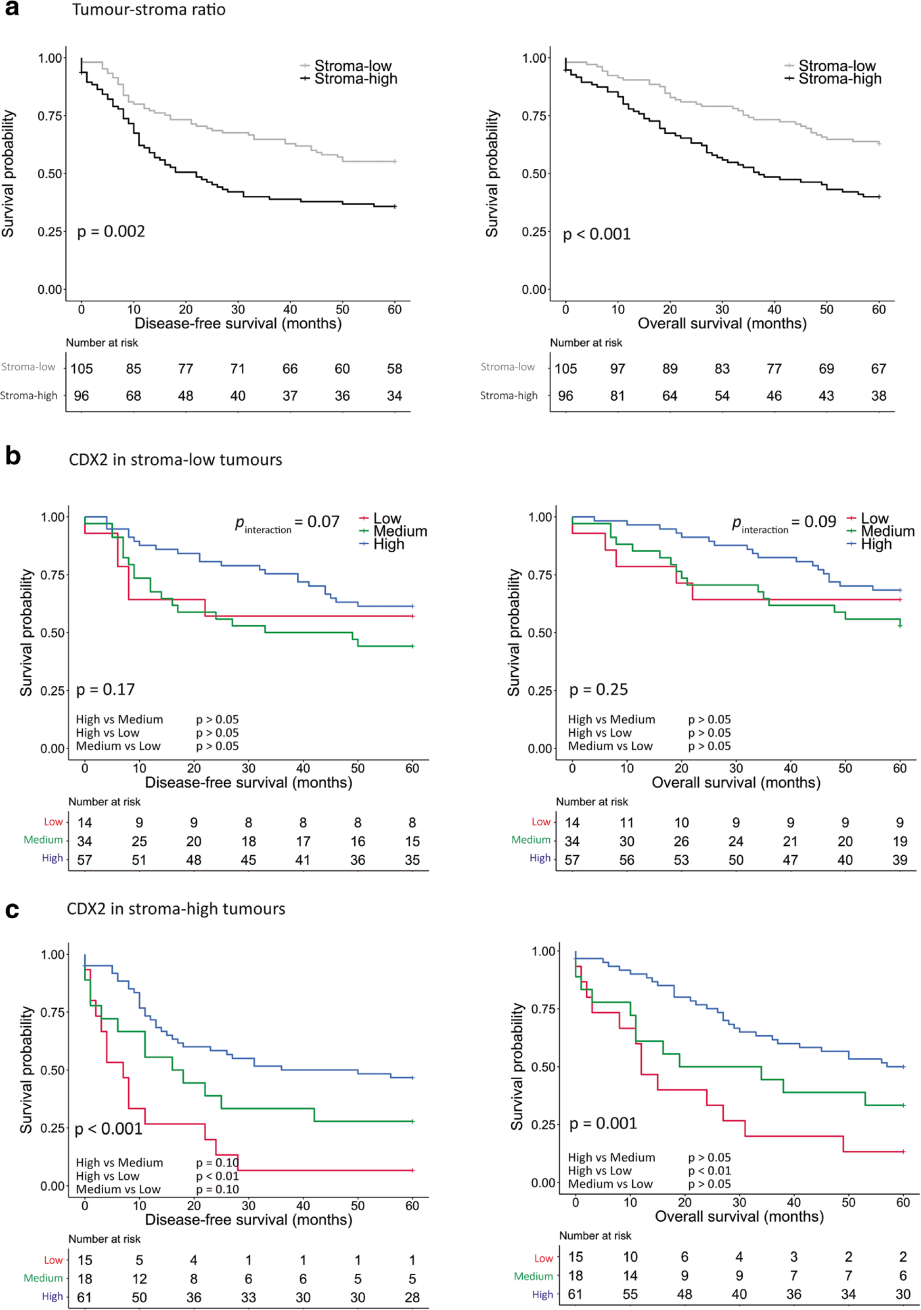
Survival of patients with CRC (*N* = 201) stratified by tumour-stroma ratio (**a**) and by CDX2 status in the stroma-low subgroup (**b**; *N* = 105) and the stroma-high subgroup (**c**; *N* = 96) (log-rank test, pairwise comparisons controlling for false discovery rate using the Benjamini-Hochberg procedure and unadjusted *p* value for interaction between tumour-stroma ratio and CDX2 status in disease-free survival and overall survival)

The TSR analysis resulted in 5-year DFS and OS rates of 55.2% and 66% in the stroma-low group, and 35.8% and 39% in the stroma-high group, respectively (Fig. 2a). The stroma-high group had a significantly worse DFS and OS compared to the stroma-low group [DFS_multivariate_*p* = 0.028, HR 1.52 (95% CI 1.05–2.21); OS_multivariate_*p* = 0.049, HR 1.43 (95% CI 1.00–2.04)] (Table 1). CDX2 status revealed 5-year DFS and OS rates of 54.2% and 59.3% in the CDX2 high group, 38.5% and 46.2% in the CDX2 medium group, and 31.0% and 37.9% in the CDX2 low group, respectively. CDX2 low cases had a worse DFS in the univariate analysis [*p* = 0.037, HR 1.66 (95% CI 1.03–2.67) CDX2-high versus CDX2-low; *p* = 0.339, HR 1.22 (0.82–1.81) CDX2-high versus CDX2-medium] and in the multivariate analysis [*p* = 0.012, HR 1.93 (95% CI 1.16–3.23) CDX2-high versus CDX2-low; *p* = 0.089, HR 1.46 (95% CI 0.95–2.26) CDX2-high versus CDX2-medium] after adjusting for TNM stage, age and adjuvant chemotherapy (Supplementary Tables 2 and 3). However, no significant difference was observed in OS [OS_univariate_*p* = 0.158, HR 1.43 (95% CI 0.87–2.34) CDX2-high versus CDX2-low; *p* = 0.300, HR 1.24 (95% CI 0.83–1.85) CDX2-high versus CDX2-medium] (Supplementary Table 2).

**Table 1:** Multivariate analysis in a CRC cohort stratified by tumour-stroma ratio

	Disease-free survival (*N* = 182)		Overall survival (*N* = 199)	
	HR (95% CI)	*Pp* value	HR (95% CI)	*Pp* value
Tumour-stroma ratio				
Stroma-low	1.00	**0.03**	1.00	0.05
Stroma-high	1.52 (1.05–2.21)		1.43 (1.00–2.04)	
Covariables *				
TNM stage				
Stage I and II	NA**	NA**	NA**	NA**
Stage III				
Stage IV				
Age				
< 50	1.00		1.00	
50–60	1.10 (0.40–2.88)	0.89	0.93 (0.36–2.40)	0.89
60–70	2.61 (1.08–6.28)	**0.03**	2.60 (1.14–5.91)	**0.02**
>70	3.44 (1.46–8.08)	**< 0.01**	4.10 (1.86–9.05)	**< 0.01**
Adjuvant therapy				
Yes	1.00	**0.03**	–	
No	0.54 (0.31–0.94)			

*N* number of patients, *HR* hazard ratio, *95% CI* 95% confidence interval, *NA* not available, *p* < 0.05 are in bold

*The multivariate analysis was adjusted for the covariables selected based on backward selection using the AIC score

**Stratification of TNM due to violation of proportional hazards assumption

Next, we assessed whether the effect of CDX2 status was different in the stroma-high and stroma-low groups. We found that within the stroma-low group, the DFS and OS rates of the CDX2-low subgroup (13.3% CDX2 low in stroma-low group) did not differ compared to those in the CDX2 high subgroup [DFS_multivariate_*p* = 0.229, HR 1.64 (0.73–3.64); OS_multivariate_*p* = 0.582, HR 1.27 (0.54–2.97)] (Fig. 2b, Table 2). Within the stroma-high group, the CDX2 low group (16.1% CDX2 low in stroma-high group) showed a 5-year DFS and a 5-year OS of 6.7% and 13.3%, respectively (Fig. 2c, Supplementary Table 1). The CDX2 low subgroup had a significant lower DFS and a borderline significant lower OS compared to the CDX2 high subgroup [DFS_multivariate_*p* = 0.002, HR 3.02 (95% CI 1.49–6.13); OS_multivariate_*p* = 0.053; HR 1.98 (95% CI 1.00–3.96)] (Table 2). The test for interaction between TSR and CDX2 status in the univariate analysis proved to be borderline significant (DFS_univariate_*p* = 0.071; OS_univariate_*p* = 0.090). The CDX2 status was compared in stage I-III CRC patients with stroma-high tumours. The Kaplan-Meier curves showed a difference in DFS (log-rank test *p* = 0.025) between cases with different CDX2 intensities (Supplementary Fig. 2). However, patients with CDX2 medium and CDX2 low tumours appeared to have a similar OS (log-rank test *p* > 0.05).

**Table 2:** Multivariate analysis in a CRC cohort stratified by interaction between tumour-stroma ratio and CDX2 status

	Disease-free survival (*N* = 180)		Overall survival (*N* = 180)	
	HR (95% CI)	*Pp* value	HR (95% CI)	*pP* value
		0.29*		0.17*
CDX2 within stroma-low				
CDX2 - high	1.00	0.26	1.00	0.42
CDX2 - medium	1.42 (0.78–2.58)	0.26	1.28 (0.71–2.32)	0.42
CDX2 - low	1.64 (0.73–3.64)	0.23	1.27 (0.54–2.97)	0.58
CDX2 within stroma-high				
CDX2 - high	1.00		1.00	
CDX2 - medium	2.61 (1.33–5.10)	**< 0.01**	3.00 (1.53–5.87)	**< 0.01**
CDX2 - low	3.02 (1.49–6.13)	**< 0.01**	1.98 (1.00–3.96)	**0.05**
Tumour-stroma ratio				
Stroma-low	1.00	0.26	1.00	0.42
Stroma-high	1.32 (0.81–2.16)		1.23 (0.74–2.04)	
Covariables**				
TNM stage				
Stage I and II	1.00		NA***	NA***
Stage III	2.14 (1.33–3.44)	**< 0.01**		
Stage IV	6.23 (3.79–10.28)	**< 0.01**		
Age				
< 50	1.00		1.00	
50–60	0.85 (0.30–2.35)	0.75	0.90 (0.29–2.74)	0.85
60–70	3.06 (1.26–7.41)	**0.01**	3.72 (1.42–9.75)	**0.02**
>70	3.47 (1.48–8.12)	**< 0.01**	5.15 (2.02–13.16)	**< 0.01**
Adjuvant therapy				
Yes	1.00	**< 0.01**	1.00	0.14
No	0.46 (0.26–0.82)		0.65 (0.36–1.15)	
Sex				
Female	1.00	0.18	–	
Male	1.29 (0.89–1.88)			

*N* number of patients, *HR* hazard ratio, *95% CI* 95% confidence interval, *NA* not available, *p *< 0.05 are in bold

*p value for interaction

**The multivariate analysis was adjusted for the covariables selected based on backward selection using the AIC score

***Stratification of TNM due to violation of proportional hazards assumption

The present study confirms the prognostic value of stroma-high tumours assessed based on the histological TSR. Furthermore, by combining stromal content and nuclear CDX2 expression, we identified a subgroup of patients with an extremely poor prognosis. We found that patients with tumours classified as stroma-high and CDX2 low had a 5-year DFS rate of 7%. However, only a borderline statistical interaction was observed between the TSR and CDX2 status. Therefore, we cannot conclude that there is a difference in stroma-low and stroma-high groups with respect to CDX2 status and survival. An explanation for this may be that the CDX2 low cases in the stroma-low group represented a small number of patients, thereby resulting in a lack of power in the analysis. From a biological perspective, it is known that the CDX2 expression level may affect the aggressive behaviour of cancer cells. Several experimental studies have shown that the tumour stroma can affect CDX2 expression in cancer cells, leading to invasion and metastatic growth [14, 15].

In accordance with previous reports [1–6], CDX2 expression was found to be prognostic in the present study. A rather small proportion (14.6%) of the CRC cohort had CDX2 low tumours. This is in line with the validation set of Dalerba et al. [4] and with the discovery set of Pilati et al. [6] in which CDX2 expression was assessed using transcriptomic data. CDX2 negative cases were less frequently noted in the discovery dataset and in the pooled dataset using immunohistochemistry in both studies. Furthermore, Pilati et al. [6] identified CDX2 low tumours exclusively in CMS1 and high stromal CMS4 subtypes, while in the present study CDX2 low tumours were also found in MSS stroma-low tumours. Nolte et al. [5] did not observe any differences in staining intensity between the invasive and centre regions. These observations suggest that TMAs can reliably be used to score CDX2 as a prognostic biomarker. A main limitation of the present study is the relatively small heterogeneous cohort used, including patients with stage I – IV disease. The multivariate analysis, for instance, could not be adjusted for pathological differentiation grade due to a high number of missing values. Although the sensitivity analysis confirmed a poor prognosis of low CDX2 expression in stroma-high tumours (stage I – III CRC), the results should be validated in a large homogenous cohort. Regarding the CDX2 scoring method, a high inter-agreement suggests that the observers could readily distinguish CDX2 low staining from CDX2 medium and high staining. However, the method may be objectivated by scoring the staining intensity automatically. The present study adhered to the REMARK guidelines to ensure transparent and complete reporting of the study (Supplementary Table 4) [16].

Retrospective studies have shown that tumours with a high stromal content do not respond to chemotherapy nor to anti-EGFR treatment independent of *RAS* mutation status [10, 17]. Therefore, studies are underway to identify targeted therapeutic options beneficial for the CMS4 subtype (rectal adenocarcinoma NCT02688712, phase II and tyrosine kinase inhibitor [18]). Once a beneficial treatment for this subgroup will be available, an easy to use stratification method will be necessary. Combining the TSR with CDX2 expression using solely microscopic analysis is technically simple to implement in pathology routine and could, therefore, serve as a useful tool to identify MSS patients with a particularly poor prognosis for whom appropriate targeted therapy would be applicable.

## Electronic supplementary material

ESM 1 (DOCX 629 kb)

## Abbreviations

CDX2: Caudal type homeobox 2
CMS: Consensus molecular subtype
CRC: Colorectal cancer
DFS: Disease-free survival
H&E: Haematoxylin and eosin
HR: Hazard ratio
IHC: Immunohistochemistry
MSI: Microsatellite instable
MSS: Microsatellite stable
OS: Overall survival
TMA: Tissue microarray
TSR: Tumour-stroma ratio
