# Supplementary material for: Prognostic value of low CDX2 expression in colorectal cancers with a high stromal content – a short report
Source: Cell Oncol (Dordr). 2019 Mar 8;42(3):397–403. doi: 10.1007/s13402-019-00436-0 (PMC12994344; doi:10.1007/s13402-019-00436-0)
Supplement: Supplementary file 1 — (DOCX 629 kb) [file 13402_2019_436_MOESM1_ESM.docx]

**Prognostic value of low CDX2 expression in colorectal cancers with a high stromal content – a short report**

T.P. Sandberg^1,2, *^, Iris Sweere^2^, Gabi W. van Pelt^1^, Hein Putter^3^, Louis Vermeulen^4^, Peter J. Kuppen^1^, Rob A.E.M. Tollenaar^1^, Wilma E. Mesker^1^

^1^ Department of Surgery, Leiden University Medical Centrum, the Netherlands

^2^ Department of Pathology, Leiden University Medical Centrum, the Netherlands

^3^ Department of medical statistics, Leiden University Medical Centrum, the Netherlands

^4^ Laboratory for Experimental Oncology and Radiobiology (LEXOR), Center for Experimental and Molecular Medicine (CEMM), Academic Medical Center & Cancer Center Amsterdam, Amsterdam, The Netherlands.

* Corresponding author t.p.sandberg@lumc.nl

**Supplementary information**

**Overview**

1. **Supplementary figures**
   **Fig S1** Flow chart of included samples  **Fig S2** Sensitivity analysis in stage I – III colorectal cancer patients
2. **Supplementary table
   Table S1** Patient characteristics stratified by the tumour-stroma ratio
   **Table S2** Univariate analysis of variables
   **Table S3** Multivariate analysis stratified by CDX2 status
   **Table S4** The REMARK checklist
3. **Supplementary figures**

LUMC cohort

N = 240

Total cohort

N = 236

Missing H&E for TSR
N = 4

CDX2 and TSR cohort

N = 199

QC CDX2 IHC

N = 2

MSS

N = 201

MSI +

N = 35

**Fig S1** Flowchart of included samples. Omitted patients are in red boxes.


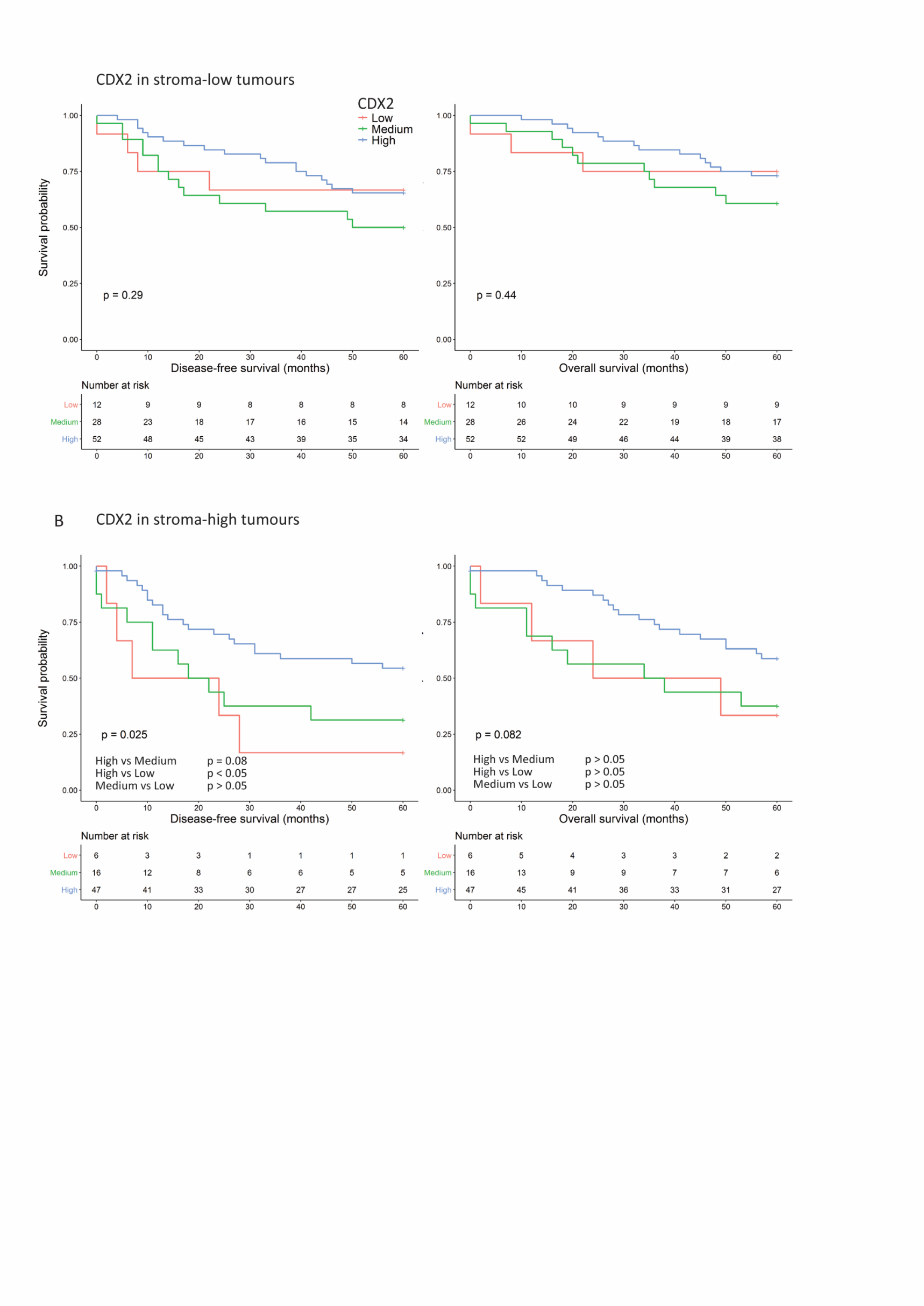


**Fig S2** Sensitivity analyses in stage I – III colorectal cancer patients in stroma-low (A) and stroma-high (B) (log-rank test, pairwise comparisons controlling for false discovery rate using Benjamini-Hochberg procedure).

1. **Supplementary tables
   Table S1** – Patient characteristics stratified by the tumour-stroma ratio.

|  |  |  | **Tumour-stroma ratio** | |
| --- | --- | --- | --- | --- |
|  |  | **Total (%) N = 201** | **Stroma L (%) N = 105 (52.2)** | **Stroma H (%) N = 96 (47.8)** |
| **CDX2** | |  |  |  |
|  | CDX2 - high | 118 (59.3) | 57 (54.3) | 61 (64.9) |
|  | CDX2 - medium | 52 (26.1) | 34 (32.4) | 18 (19.2) |
|  | CDX2 - low | 29 (14.6) | 14 (13.3) | 15 (16.1) |
|  | NA | 2 | 0 | 2 |
|  |  |  |  |  |
| **Median age at surgery** (range) | | 68.18 (31 – 91) | 67.91 (31 – 90) | 68.51 (35 – 91) |
|  |  |  |  |  |
| **Sex** | |  |  |  |
|  | Male | 102 (50.7) | 52 (49.5) | 50 (52.1) |
|  | Female | 99 (49.3) | 53 (50.5) | 46 (47.9) |
|  |  |  |  |  |
| **TNM stage (AJCC 5)** | |  |  |  |
|  | I | 33 (16.4) | 22 (21.2) | 11 (11.6) |
|  | II | 71 (35.3) | 38 (36.5) | 33 (34.7) |
|  | III | 59 (29.4) | 32 (30.8) | 27 (28.4) |
|  | IV | 36 (17.9) | 12 (11.5) | 24 (25.3) |
|  | NA | 2 | 1 | 1 |
|  |  |  |  |  |
| **Tumour site** | |  |  |  |
|  | Colon | 159 (79.1) | 77 (74.0) | 82 (86.3) |
|  | Rectum | 40 (19.9) | 27 (26.0) | 13 (13.7) |
|  | NA | 2 | 1 | 1 |
|  |  |  |  |  |
| **Differentiation** | |  |  |  |
|  | G1 well | 38 (18.9) | 18 (24.0) | 20 (25.0) |
|  | G2 moderate | 104 (51.7) | 53 (70.7) | 51 (63.8) |
|  | G3 poor | 13 (6.5) | 4 (5.3) | 9 (11.3) |
|  | NA | 46 (22.9) | 30 (28.6) | 16 (16.7) |
|  |  |  |  |  |
| **Adjuvant therapy** | |  |  |  |
|  | Yes | 31 (15.4) | 8 (7.6) | 23 (24.0) |
|  | No | 153 (76.2) | 85 (81.0) | 68 (70.8) |
|  | NA | 17 (8.5) | 12 (11.4) | 5 (5.2) |
|  |  |  |  |  |
| **Vital status after 5 years** | |  |  |  |
|  | Alive | 105 (52.2) | 66 (62.9) | 39 (40.6) |
|  | Death | 96 (47.8) | 39 (37.1) | 57 (59.4) |
|  |  |  |  |  |
| **Median follow-up time OS** | | 68 (0 – 229) | 104 (0 – 229) | 36.5 (0 – 210) |
| (range) |  |  |  |  |
|  |  |  |  |  |

N = number of patients included, NA = data not available

**Table S2** – Univariate analysis including tumour-stroma ratio, CDX2 status, age, gender, TNM stage, tumour site, differentiation and adjuvant therapy.

|  |  | **UNIVARIATE ANALYSIS** | | | |
| --- | --- | --- | --- | --- | --- |
|  |  | **Disease-free survival** | | **Overall survival** | |
|  |  | HR (95% CI) | P-value | HR (95% CI) | P-value |
| **Tumour-stroma ratio** | |  |  |  |  |
|  | Stroma-low | 1.00 | **< 0.01** | 1.00 | **< 0.01** |
|  | Stroma-high | 1.79 (1.28 – 2.51) |  | 1.78 (1.26 – 2.51) |  |
|  |  |  |  |  |  |
| **CDX2 status** | |  |  |  |  |
|  | CDX2 - high | 1.00 |  | 1.00 | 0.29 |
|  | CDX2 - medium | 1.22 (0.82 – 1.81) | 0.33 | 1.24 (0.83 – 1.85) |  |
|  | CDX2 - low | 1.66 (1.03 – 2.67) | **0.04** | 1.43 (0.87 – 2.34) |  |
|  |  |  |  |  |  |
| **Age** |  |  |  |  |  |
|  | < 50 | 1.00 |  | 1.00 |  |
|  | 50 – 60 | 1.11 (0.47 – 2.61) | 0.93 | 1.18 (0.48 – 2.92) | 0.82 |
|  | 60 – 70 | 2.96 (1.39 – 6.32) | **< 0.01** | 3.29 (1.47 – 7.36) | **< 0.01** |
|  | >70 | 3.50 (1.68 – 7.29) | **< 0.01** | 4.26 (1.95 – 9.29) | **< 0.01** |
|  |  |  |  |  |  |
| **Sex** |  |  |  |  |  |
|  | Female | 1.00 | 0.24 | 1.00 | 0.25 |
|  | Male | 0.82 (0.58 – 1.14) |  | 0.82 (0.58 – 1.15) |  |
|  |  |  |  |  |  |
| **TNM stage (AJCC 5)** | |  |  |  |  |
|  | I and II | 1.00 |  | 1.00 |  |
|  | III | 1.70 (1.15 – 2.53) | **< 0.01** | 1.76 (1.17 – 2.64) | **< 0.01** |
|  | IV | 5.17 (3.33 – 8.01) | **< 0.01** | 5.44 (3.50 – 8.46) | **< 0.01** |
|  |  |  |  |  |  |
| **Tumour site** | |  |  |  |  |
|  | Colon | 1.00 | 0.55 | 1.00 | 0.30 |
|  | Rectum | 0.88 (0.57 – 1.35) |  | 0.80 (0.51 - 1.23) |  |
|  |  |  |  |  |  |
| **Differentiation** | |  |  |  |  |
|  | G1 well | 1.00 | 0.54 | 1.00 | 0.82 |
|  | G2 moderate | 1.14 (0.71 – 1.81) |  | 1.04 (0.47 – 2.33) |  |
|  | G3 poor | 1.53 (0.73 – 3.12) |  | 1.15 (0.72 – 1.85) |  |
|  |  |  |  |  |  |
| **Adjuvant therapy** | |  |  |  |  |
|  | Yes | 1.00 | 0.97 | 1.00 | 0.61 |
|  | No | 1.01 (0.63 - 1.63) |  | 0.88 (0.55 – 1.43) |  |
|  |  |  |  |  |  |

N = number of patients included in the analysis, HR = hazard ratio, 95% CI = 95% confidence interval

**Table S3**– Multivariate analysis stratified by CDX2 status.

|  | | | **Disease-free survival (N = 180)** | | | |
| --- | --- | --- | --- | --- | --- | --- |
|  | |  | |  | HR (95% CI) | P-value |
| **CDX2** | | | |  |  |  |
|  | CDX2 - low | | |  | 1.00 | 0.09 |
|  | CDX2 - medium | | |  | 1.46 (0.95 - 2.26) |  |
|  | CDX2 - high | | |  | 1.93 (1.16 – 3.23) | **0.01** |
|  |  | | |  |  |  |
| **TNM stage** | | | |  |  |  |
|  | Stage I and II | | |  | 1.00 |  |
|  | Stage III | | |  | 2.24 (1.40 – 3.58) | **< 0.01** |
|  | Stage IV | | |  | 6.11 ( 3.78 – 9.88) | **< 0.01** |
|  |  | | |  |  |  |
| **Age** | | | |  |  |  |
|  | < 50 | | |  | 1.00 |  |
|  | 50 – 60 | | |  | 0.89 (0.32 – 2.46) | 0.82 |
|  | 60 – 70 | | |  | 3.19 (1.33 – 7.64) | **< 0.01** |
|  | >70 | | |  | 3.76 (1.61 – 8.78) | **< 0.01** |
|  |  | | |  |  |  |
| **Adjuvant therapy** | | | |  |  |  |
|  | Yes | | |  | 1.00 | 0.07 |
|  | No | | |  | 0.60 (0.34 – 1.04) |  |
|  |  | | |  |  |  |

The multivariate analyse was adjusted for the covariables selected based on backward selection using the AIC score
N = number of patients included in the analysis, HR = hazard ratio, 95% CI = 95% confidence interval

**Table S4 –** The REMARK checklist

|  | | **Page no.** |
| --- | --- | --- |
| **INTRODUCTION** | |  |
| 1 | State the marker examined, the study objectives, and any pre-specified hypotheses. | p3 |
| **MATERIALS AND METHODS** | |  |
| *Patients* | |  |
| 2 | Describe the characteristics (e.g., disease stage or co-morbidities) of the study patients, including their source and inclusion and exclusion criteria. | p3 + Suppl table1 |
| 3 | Describe treatments received and how chosen (e.g., randomized or rule-based). | p3 |
| *Specimen characteristics* | |  |
| 4 | Describe type of biological material used (including control samples) and methods of preservation and storage. | p4 |
| *Assay methods* | |  |
| 5 | Specify the assay method used and provide (or reference) a detailed protocol, including specific reagents or kits used, quality control procedures, reproducibility assessments, quantitation methods, and scoring and reporting protocols. Specify whether and how assays were performed blinded to the study endpoint. | P4 - 5 |
| *Study design* | |  |
| 6 | State the method of case selection, including whether prospective or retrospective and whether stratification or matching (e.g., by stage of disease or age) was used. Specify the time period from which cases were taken, the end of the follow-up period, and the median follow-up time. | P3  P5 Table S1 |
| 7 | Precisely define all clinical endpoints examined. | P5 |
| 8 | List all candidate variables initially examined or considered for inclusion in models. | P5 |
| 9 | Give rationale for sample size; if the study was designed to detect a specified effect size, give the target power and effect size. | NA |
| *Statistical analysis methods* | |  |
| 10 | Specify all statistical methods, including details of any variable selection procedures and other model-building issues, how model assumptions were verified, and how missing data were handled. | P5 |
| 11 | Clarify how marker values were handled in the analyses; if relevant, describe methods used for cutpoint determination. | P5 |
| **RESULTS** | |  |
| *Data* | |  |
| 12 | Describe the flow of patients through the study, including the number of patients included in each stage of the analysis (a diagram may be helpful) and reasons for dropout. Specifically, both overall and for each subgroup extensively examined report the numbers of patients and the number of events. | Figure S1 |
| 13 | Report distributions of basic demographic characteristics (at least age and sex), standard (disease-specific) prognostic variables, and tumour marker, including numbers of missing values. | Table S1 |
| *Analysis and presentation* | |  |
| 14 | Show the relation of the marker to standard prognostic variables. | NA |
| 15 | Present univariable analyses showing the relation between the marker and outcome, with the estimated effect (e.g., hazard ratio and survival probability). Preferably provide similar analyses for all other variables being analyzed. For the effect of a tumour marker on a time-to-event outcome, a Kaplan-Meier plot is recommended. | Table S2  Fig 2 |
| 16 | For key multivariable analyses, report estimated effects (e.g., hazard ratio) with confidence intervals for the marker and, at least for the final model, all other variables in the model. | Table 1 - 2 |
| 17 | Among reported results, provide estimated effects with confidence intervals from an analysis in which the marker and standard prognostic variables are included, regardless of their statistical significance. | P6 - 7 |
| 18 | If done, report results of further investigations, such as checking assumptions, sensitivity analyses, and internal validation. | p. 6  Table 1 - 2 |
| **DISCUSSION** | |  |
| 19 | Interpret the results in the context of the pre-specified hypotheses and other relevant studies; include a discussion of limitations of the study. | P7-8 |
| 20 | Discuss implications for future research and clinical value. | P7-8 |
